# Metastatic Lung Adenocarcinoma: A Case of Unusual Presentation With a Skull Mass

**DOI:** 10.7759/cureus.42399

**Published:** 2023-07-24

**Authors:** Jaha Oh, Eunhee Choi, Richa Aggarwal

**Affiliations:** 1 Department of Internal Medicine, New York City (NYC) Health + Hospitals/Lincoln, Bronx, USA

**Keywords:** skull mass resection, occipital skull metastasis, adenocarcinoma lung, skull mass, bone metastasis

## Abstract

This case report describes an uncommon presentation of lung adenocarcinoma, which appeared as a skull mass. While not the first reported case in medical literature, it is still a rare occurrence for lung adenocarcinoma to present in this manner. This report focuses on the clinical presentation and treatment of an elderly male patient who had a progressively enlarging and painful skull mass. The initial imaging revealed an about 5 cm soft tissue mass at the dorsal midline of the parietal-occipital bone. Subsequent imaging identified a lung mass, and a biopsy of the skull bone confirmed that the mass was metastatic adenocarcinoma originating from the lung. For treatment, the patient underwent occipital partial resection of the mass, followed by wire mesh cranioplasty. Chemotherapy and external beam radiotherapy were administered to alleviate symptoms and control the spread of cancer. Lung carcinoma with distant metastasis is generally associated with a poorer prognosis. However, some supporting data suggest that early detection and aggressive management play crucial roles in preventing further metastasis and improving the patient's quality of life and overall survival rate. Skull bone metastasis from lung cancer is indeed a rare phenomenon, and cases like these contribute valuable knowledge to the field. By reporting such cases, healthcare professionals can gain a better understanding of the clinical manifestations, diagnostic challenges, and appropriate management strategies for these uncommon occurrences. This case report underscores the significance of maintaining a high index of suspicion and utilizing a multimodality approach to diagnose rare instances of calvarial metastasis.

## Introduction

Calvarial lesions are common in medical practice and can be either benign or malignant. Among the different possibilities, it is essential not to overlook the potential for malignancy. The skull serves as a frequent site for cancer metastases, where tumors from distant parts of the body spread through the bloodstream. These metastases often manifest as multiple lytic lesions, causing pain and involving adjacent soft tissues [1]. With an overall survival rate of only 5-11 months after diagnosing a metastatic skull tumor in patients with lung cancer [2], identifying the primary cause can prove daunting, especially when initial symptoms appear to be associated with distant metastasis.

To navigate such complexities, physicians must adopt a comprehensive approach, considering factors like the patient's age, medical history, and imaging studies to arrive at an accurate diagnosis [3]. Early detection is vital, enabling prompt initiation of systemic therapies like chemotherapy, participation in clinical trials, or palliative care, all of which play crucial roles in managing symptoms and controlling the spread of cancer to distant sites.

In this case report, we describe a case of an elderly male who presented with a headache caused by a skull mass, the initial and unexpected manifestation of lung metastasis that drew medical attention.

## Case presentation

A 68-year-old Hispanic male presented with a one-month history of a progressively enlarging and painful head mass. The patient reported accompanying headaches, which prompted him to seek medical evaluation. Upon admission, the patient provided his medical record, which included a head computed tomography (CT) scan that had been performed in his country of origin two weeks prior to his arrival at the hospital.

The CT scan revealed a soft tissue mass measuring 39 x 32 mm located at the dorsal midline of the parietal-occipital bone. However, it was noted that the patient had not reviewed the results of the CT scan before coming to the USA. His past medical history was significant for chronic obstructive pulmonary disease with a social history of 40-pack-year smoking.

Upon presentation, the patient denied having nausea, seizures, loss of consciousness, cough, or shortness of breath. On physical examination, he was vitally stable. There was a palpable, round, non-pulsatile, tender mass measuring 5 x 2 cm on the occipital area of the head. The examination of the other systems was unremarkable. Laboratory testing, which included a complete blood count, renal function test, liver function test, and serum electrolyte levels, did not yield any abnormalities.

**Figure 1:**
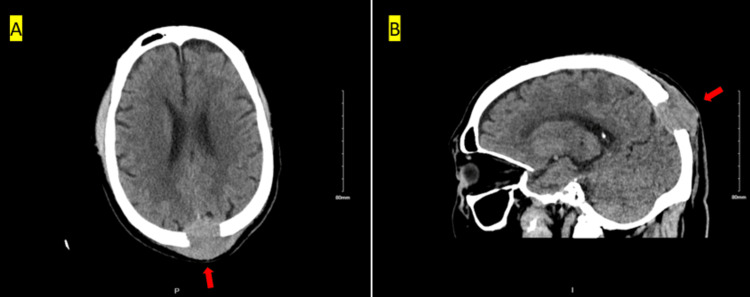
CT head without contrast 5.0 x 4.2 x 3.3 cm of soft tissue mass centered within the dorsal midline parietal-occipital bone with extension into the epidural space and invasion of the superior sagittal sinus. The mass slightly compresses the underlying parietal and occipital cortex without associated vasogenic edema.

A CT scan of the head without contrast revealed a 5 cm soft tissue mass located at the dorsal midline parietal-occipital bone (Figures 1A, 1B). The mass was observed to extend into the epidural space and invade the superior sagittal sinus. Given the rapid growth of the head mass and the imaging findings, our initial differential diagnosis considered the possibilities of metastatic disease and multiple myeloma. Serum protein electrophoresis (SPEP) was negative. Consequently, the patient was started on levetiracetam to prevent seizures and referred to the neurosurgery department for further evaluation and management. 

**Figure 2:**
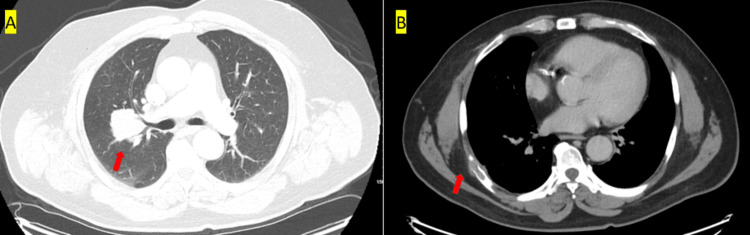
CT chest with contrast (A) 4.8 x 4.2 x 4.6 cm lobulated solid mass right upper lobe contiguous with the pulmonary hila. (B) Lytic lesion posteriorly right 7th rib with pathologic fracture.

**Figure 3:**
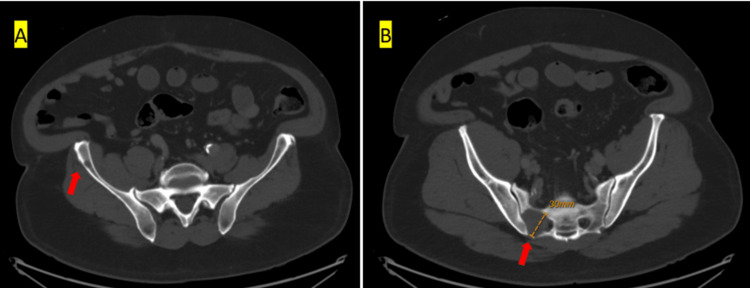
CT abdomen pelvis with contrast (A) Small lytic lesion in the right iliac wing, and 3.5 cm lytic lesion. (B) Right sacral ala without fracture

**Figure 4:**
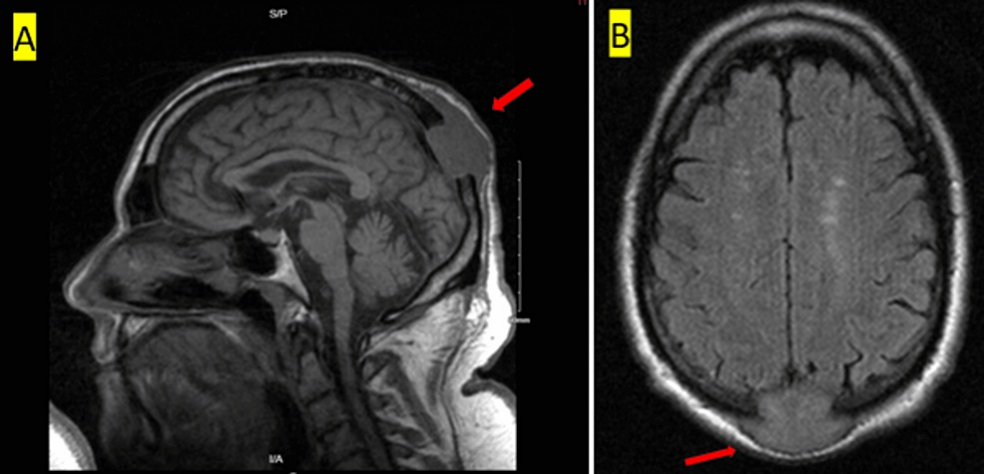
MRI brain without contrast (A) Anterior displacement of the superior sagittal sinus, without evidence of sinus invasion. (B) 5 cm lytic mass centered in the posterior midline parietal and occipital bone eroding through the inner and outer table. Mild extension into the epidural space with mild mass effect on the parietal lobe.

To assess for metastasis, a comprehensive CT scan of the chest, abdomen, and pelvis with contrast was performed. The scan revealed a lobulated solid mass measuring 4.8 x 4.2 x 4.6 cm in the right upper lobe of the lung (Figure 2A), along with a lytic lesion and pathologic fracture on the posterior aspect of the right 7th rib, a 3 cm soft tissue mass over the right 11th rib (Figure 2B), a small lytic lesion on the right iliac wing (Figure 3A), and on the right sacral ala (Figure 3B). There were no remarkable abnormalities in other internal organs such as thyroid glands, prostate, or kidney on the CT scans. An MRI of the brain without contrast was conducted to see surrounding structure involvement. It showed a lytic mass centered in the posterior midline parietal and occipital bone, measuring approximately 5.1 x 2.6 x 4.0 cm (craniocaudal by anteroposterior by transverse) (Figures 4A, 4B). The mass had eroded through the inner and outer tables of the skull, extending into the epidural space and overlying subcutaneous fat.

As a result, the patient was referred to interventional radiology for a biopsy of the skull mass. The day after the biopsy, the patient underwent occipital mass resection with wire mesh cranioplasty. Afterward, immunohistology revealed positive CK7 and CK 20 staining and negative TTF1 staining with PD-L1, suggesting a lung origin of metastatic adenocarcinoma. Next-generation sequencing was remarkable for a co-mutation of KRAS and STK11. The patient's lung cancer was staged as Stage IVB (cT2b, cN0, pM1c) based on the American Joint Committee on Cancer (AJCC) 8th Edition criteria. Given that the patient has untreated pulmonary mass with multiple bone metastases without brain parenchymal invasion, a decision was made to provide systemic chemotherapy prior to offering palliative external beam radiotherapy in order to treat the underlying disease and potentially reduce the overall tumor burden. However, after the first cycle of pembrolizumab, carboplatin, and pemetrexed, the patient developed neutropenia. The patient refused further chemotherapy and radiation therapy and returned to his home country, leading to the loss of follow-up.

## Discussion

Soft tissue masses in the head can be linked to infections, benign tumors, and malignant tumors [4]. Certain features such as a large size (>5 cm), sudden onset or rapid growth, firm consistency, and adherence to surrounding structures raise concerns about the possibility of malignancy [5]. In the case presented here, considering the patient's extensive smoking history, age, and the rapid growth rate of the skull lesion with a significant size of 5 cm and firm characteristics, our suspicions lean more toward malignant lesions. Consequently, additional investigations were undertaken, including further imaging studies and a biopsy.

Skull malignant lesions encompass metastases, multiple myeloma, osteosarcoma, chordoma, and chondrosarcoma [5]. Each malignant skull lesion has specific imaging characteristics (Table 1) [1]. Considering that osteosarcoma typically displays an aggressive periosteal reaction, and both chordoma and chondrosarcoma commonly involve the skull base, we narrowed down our list of possible diagnoses to include metastases and multiple myeloma, the latter of which can manifest as a solitary plasmacytoma [1]. Given negative SPEP results, we suspect metastases to be more likely.

**Table 1:** Salient features of malignant skull lesions on CT and MRI

Skull lesion	Location in the skull	CT	MRI
Multiple myeloma	Calvarium, rarely in skull base	Osteolytic lesions without sclerotic rim, “punched-out lesions”.	Hypointense T1, hyperintense T2. “Salt and pepper marrow infiltration” is the most common pattern. Typically enhance.
Osteosarcoma	Most frequently in the calvarium then the skull base.	Lytic lesion, ill-defined borders. Aggressive periosteal reaction. Variable amount of osteoid matrix.	Variable appearance.
Metastases	Any layer of the skull	Lytic, sclerotic, or mixed depending on the primary tumor.	Hypo/isointense T1, hyperintense T2. Typically enhance (unless sclerotic).
Chordoma	Skull base, midline	Lytic destructive expansile lesion of the clivus.	Hypointense T1, lobules of high signal on T2. Heterogeneous enhancement.
Chondrosarcoma	Skull base, paramidline	Osteolytic lesion. Chondroid matrix with “rings and arcs”.	Hypo/isointense T1, hyperintense T2. “Whorls of enhancement”.

Metastases are the most common malignant bone tumors in adults and are usually diagnosed in the context of a known primary tumor [6]. Bone metastases are frequently secondary to breast (54.9%), lung (14.3%), prostate (6.3%), malignant lymphoma (5.1%), kidney, and thyroid cancers in adults [2]. However, in rare cases, they may represent the initial manifestation of an unknown cancer. In our case, the skull metastasis was secondary to lung adenocarcinoma which was the first presenting sign of lung cancer. This occurrence is rare, with only a few reported cases in the literature [7-10]. Among primary lung cancer cases, the most prevalent histological types were adenocarcinoma (58%), followed by small cell carcinoma (18%), squamous cell carcinoma (8%), large cell carcinoma (8%), and unknown (8%) [2].

The definitive diagnosis is only available via histopathological examination coupled with immunohistochemistry (also known as tissue diagnosis) [2]. In this case, the immunohistochemistry of the biopsy from the skull mass showed positive CK7, negative TTF1, and negative CK 20, suggesting the possibility of metastatic adenocarcinoma [11]. However, a negative TTF1 result does not definitively confirm its origin in the lung [11]. To further investigate potential primary origins, additional markers such as CDX2, NKX3.1, and PODXL-1 were stained during the surgical pathology process, aiming to explore the possibility of the colon, prostate, or kidney as the primary source [12]. The results were inconclusive in determining the specific site of origin. Therefore, a Tumor Board review was conducted, and the diagnosis of metastatic adenocarcinoma originating from the lung was confirmed through clinical and radiographic correlation. This decision took into account that lung adenocarcinoma can present with negative TTF-1 and that there were no other identifiable primary lesions [11].

According to the current NCCN guidelines, for the staging of lung cancer, a brain MRI with contrast and an FDG PET/CT scan for pre-treatment evaluation are recommended. Additionally, there is evidence that FDG-PET scanning has been shown to alter the management of approximately 20-30% of patients, primarily by upgrading the disease stage and reducing the occurrence of unnecessary thoracotomies by half [13]. However, the guidelines do not advocate the use of PET scanning for clinical Stage IV patients who already have confirmed disseminated distant metastatic disease. In our case, an MRI without contrast was conducted, because this was not done for staging cancer but as part of a pre-surgical evaluation for neurosurgery. 

Regarding bone metastases, approximately 20-30% of NSCLC patients present with bone metastases at the time of diagnosis known as synchronous bone metastasis (SBM), while 35-60% of them develop it during the course of the disease, termed metachronous bone metastasis (MBM) [2,6]. Compared to lung cancer patients with MBM, patients with SBM often exhibit a significant tumor burden and complex physiological disruptions [14]. Median survival for SBM patients is reported to be between 5 and 11 months [2,7,15]. As such, it is crucial for clinicians to remain vigilant in detecting suspected malignant skull lesions as early as possible and to provide aggressive treatments to enhance the patient's quality of life. Early detection and proactive intervention can lead to better outcomes and improved well-being for these patients.

The most common sites of metastasis in lung cancer are the spine, pelvis, and ribs, accounting for the majority of cases [16]. However, skull involvement is relatively infrequent and observed in only about 3% of cases [8]. In the context of bone metastasis in lung cancer, adhesion molecules, particularly vascular cell adhesion protein-1, play a significant role in facilitating interactions between lung cancer cells and bone cells. This interaction initiates changes in the bone microenvironment [17] which activates osteoclasts, leading to bone degradation [16], and then to pathological fractures. It can also result from metastatic bony deposits, known as skeletal-related events [18]. These events can significantly impact the patient's quality of life due to severe bone pain and require appropriate management and intervention to alleviate pain and prevent further complications.

Generally, the treatment modalities for patients with skull metastasis include irradiation, chemotherapy, targeted therapy, bisphosphonate, and surgical removal [19]. Notably, patients exhibiting signs of dura infiltration and related neurological deficits should be offered neurosurgical therapy and then should be followed by chemotherapy for primary lung lesions [19]. In case total excision of the lesion is not possible, radiotherapy is another option for treatment as illustrated in this case [7]. Although surgical removal can be challenging, especially if vital structures such as the dural sinuses are involved [7], early surgical removal is often recommended due to the infiltrative progression of tumors in the calvaria, and postoperative radiotherapy and chemotherapy may also be beneficial [15].

Despite lung carcinoma with distant metastasis being recognized as a poor predictor of survival [10], numerous reports have shown that patients with solitary metastasis experienced an improved rate of survival when the metastatic lesion was excised and treated with aggressive chemotherapy and radiotherapy [7-9]. Even in cases of multiple site metastasis, early detection and surgical removal of metastatic skull lesions are suggested to prevent or delay the cancer's further progression to other locations [9].

## Conclusions

Being vigilant and thorough in the evaluation of soft tissue masses in the head region, especially in patients with concerning symptoms, can lead to early diagnosis and timely intervention. Early detection and aggressive treatment approaches may improve survival rates in cases of solitary metastasis, and prompt management is particularly crucial in the context of rapidly growing skull metastases of lung cancer.

This specific case report highlights the rare occurrence of lung adenocarcinoma presenting as skull metastasis, contributing valuable knowledge to understand cancer's diverse manifestations. Reporting such cases is crucial to enhance medical understanding and address diagnostic challenges in advanced-stage lung cancer. 
